# Supplementary material for: Ancient and modern anticonvulsants act synergistically in a KCNQ potassium channel binding pocket
Source: Nat Commun. 2018 Sep 21;9:3845. doi: 10.1038/s41467-018-06339-2 (PMC6155021; doi:10.1038/s41467-018-06339-2)
Supplement: Supplementary file 1 — Supplementary Information [file 41467_2018_6339_MOESM1_ESM.pdf]

Ancient and modern anticonvulsants act synergistically in a KCNQ potassium channel binding pocket

Manville et al.

KCNQ2/3	Normalized tail current $V_{0.5}$ (mV)	Non-normalized tail current $V_{0.5}$ (mV)	Slope (mV)
**Ctrl**	-41.9 ± 0.3 (n =5)	-42.0 ± 4.7 (n =5)	6.5 ± 0.3 (n =5)
**100 μM 3-ethyl-2-hydroxy-2-cyclopenten-1-one**	-41.0 ± 0.9 (n =5)	-40.1 ± 1.9 (n =5)	5.1 ± 0.9 (n =5)

Supplementary Table 1. Summary of Effects of 3-ethyl-2-hydroxy-2-cyclopenten-1-one on KCNQ2/3 channels.

Values indicate mean ± SEM.

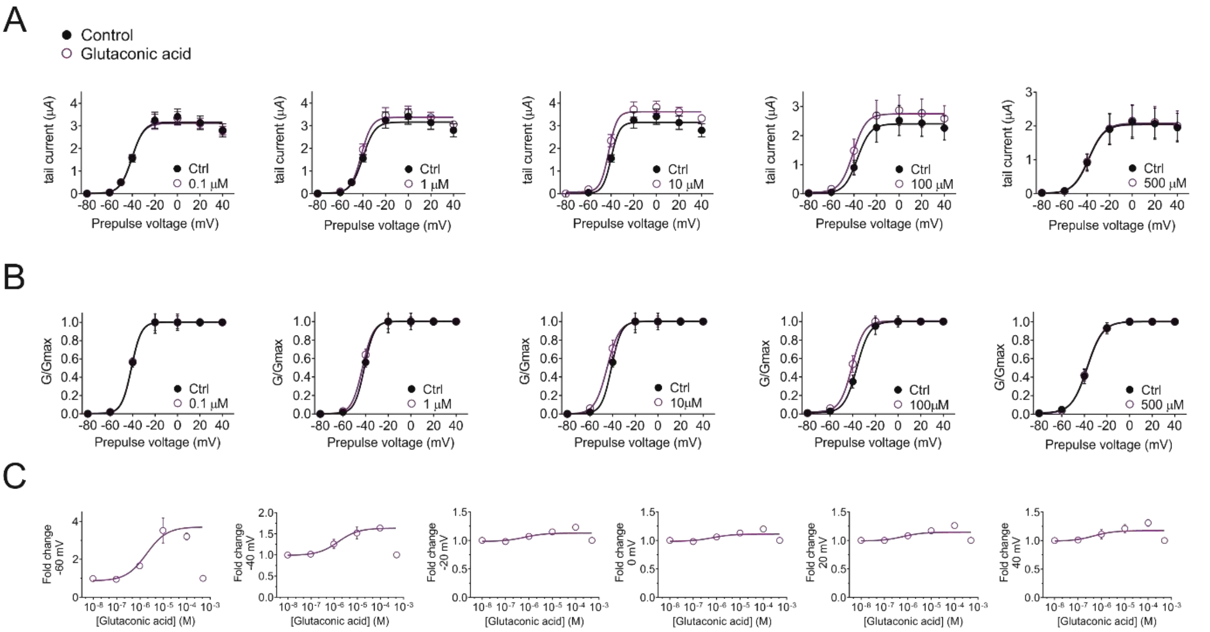

Supplementary Figure 1. Effects of Glutaconic acid on KCNQ2/3 channels

A. Mean tail current versus prepulse voltage relationship for KCNQ2/3 channels in the absence (black) and presence (purple) of Glutaconic acid, *n* = 5.

B. Normalized tail current versus prepulse voltage relationships as in panel A, *n* = 5.

C. Dose response of KCNQ2/3 channels between -40 and +40 mV, *n* = 5. Error bars indicate SEM.

**KCNQ2/3**	**Normalized tail current $V_{0.5}$ (mV)**	**Non-normalized tail current $V_{0.5}$ (mV)**	**Slope (mV)**
**Ctrl**	-36.9 ± 1.7 (n =5)	-36.1 ± 1.7 (n =5)	4.4 ± 0.5 (n =5)
**0.1 µM Glutaconic Acid**	-41.2 ± 1.4 (n =5)	-40.4 ± 1.5 (n =5)	4.4 ± 3.7 (n =5)
**1 µM Glutaconic Acid**	-42.7 ± 1.7 (n =5)	-41.5 ± 1.3 (n =5)	4.8 ± 2.4 (n =5)
**10 µM Glutaconic Acid**	-42.9 ± 2.2 (n =5)	-42.1 ± 3.8 (n =5)	5.4 ± 2.3 (n =5)
**100 µM Glutaconic Acid**	-40.7 ± 1.2 (n =5)	-40.8 ± 3.8 (n =5)	5.4 ± 1.7 (n =5)
**500 µM Glutaconic Acid**	-37.7 ± 0.9 (n =5)	-38.5 ± 5.4 (n =5)	6.8 ± 0.9 (n =5)

Supplementary Table 2. Summary of Effects of Glutaconic acid on KCNQ2/3 channels.

Values indicate mean ± SEM.

**KCNQ2/3**	**Normalized tail current $V_{0.5}$ (mV)**	**Non-normalized tail current $V_{0.5}$ (mV)**	**Slope (mV)**
**Ctrl**	41.8 ± 0.9 (n =5)	-42.0 ± 5.1 (n =5)	5.7 ± 1.6 (n =5)
**100 μM 1-Heptene**	-41.8 ± 0.9 (n =5)	-41.9 ± 4.9 (n =5)	5.8 ± 1.5 (n =5)

Supplementary Table 3. Summary of Effects of 1-Heptene on KCNQ2/3 channels.

Values indicate mean ± SEM.

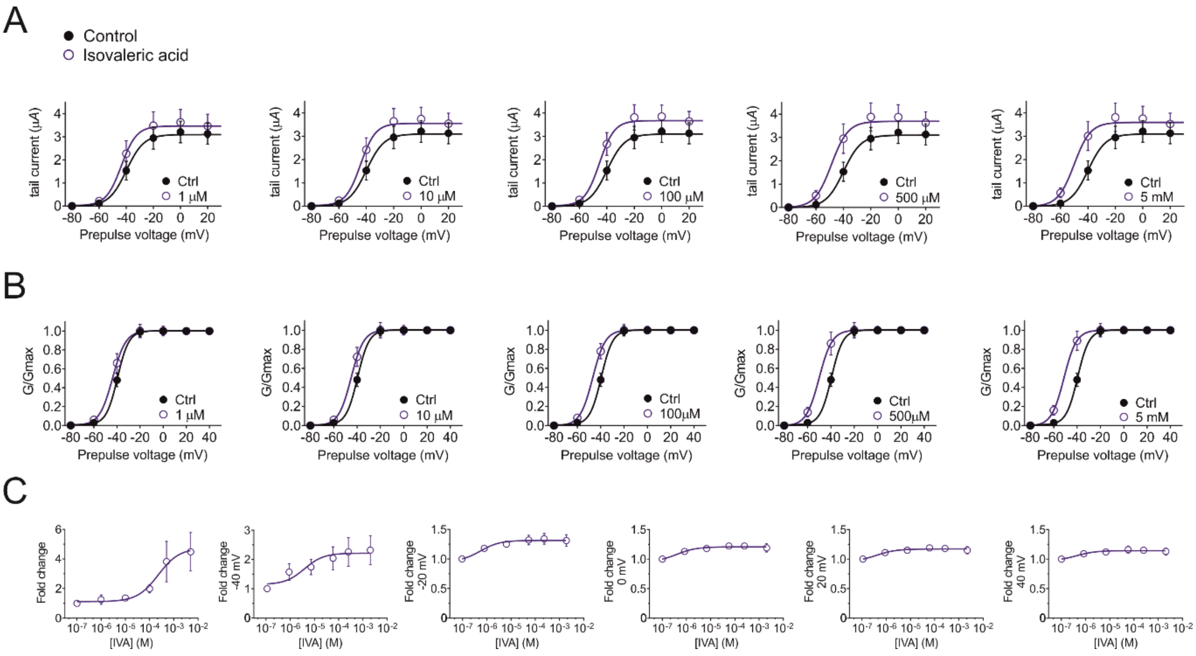

Supplementary Figure 2. Effects of Isovaleric acid (IVA) on KCNQ2/3 channels

A. Mean tail current versus prepulse voltage relationship for KCNQ2/3 channels in the absence (black) and presence (blue) of Isovaleric acid, *n* = 4.

B. Normalized tail current versus prepulse voltage relationships as in panel A, *n* = 4.

C. Dose response of KCNQ2/3 channels between -40 and +40 mV, *n* = 4. Error bars indicate SEM.

**KCNQ2/3**	**τ act, -40 mV (ms)**	**τ deact, -80 mV (ms)**	**Normalized tail current $V_{0.5}$ (mV)**	**Non-normalized tail current $V_{0.5}$ (mV)**	**Slope (mV)**
**Ctrl**	621 ± 95 (n =4)	146 ± 11 (n =4)	-39.5 ± 0.8 (n =4)	-39.9 ± 3.5 (n =4)	4.9 ± 2.2 (n =4)
**1 µM IVA**	494 ± 48 (n =4)	159 ± 12 (n =4)	-43.8 ± 1.9 (n =4)	-43.6 ± 5.1 (n =4)	5.7 ± 2.1 (n =4)
**10 µM IVA**	474 ± 37 (n =4)	170 ± 6 (n =4)	-45.1 ± 2.2 (n =4)	-44.2 ± 4.8 (n =4)	5.4 ± 1.8 (n =4)
**100 µM IVA**	442 ± 22 (n =4)	192 ± 14 (n =4) *	-46.8 ± 1.8 (n =4)	-45.4 ± 4.7 (n =4)	5.4 ± 1.1 (n =4)
**500 µM IVA**	406 ± 18 (n =4)	219 ± 21 (n =4) *	-50.1 ± 2.4 (n =4) *	-48.7 ± 5.2 (n =4)	5.6 ± 1.2 (n =4)
**5 mM IVA**	414 ± 23 (n =4)	241 ± 22 (n =4) *	-51.2 ± 2.1 (n =4) **	-50.1 ± 5.2 (n =4)	5.4 ± 1.1 (n =4)

Supplementary Table 4. Summary of Effects of IVA on KCNQ2/3 channels.

Statistics versus same channel in absence of IVA: **p=0.007, *p=0.01. Values indicate mean ± SEM.

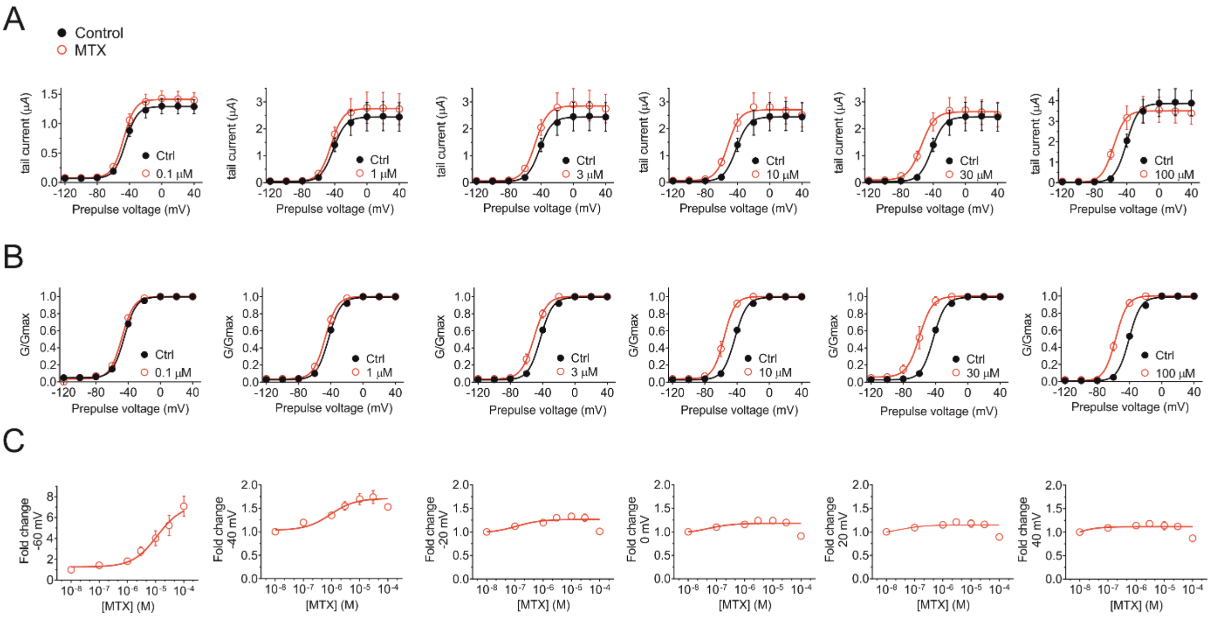

Supplementary Figure 3. Effects of MTX on KCNQ2/3 channels

A. Mean tail current versus prepulse voltage relationship for KCNQ2/3 channels in the absence (black) and presence (red) of MTX, *n* = 5-9.

B. Normalized tail current versus prepulse voltage relationships as in panel A, *n* = 5-9.

C. Dose response of KCNQ2/3 channels between -40 and +40 mV, *n* = 5-9. Error bars indicate SEM.

KCNQ2/3	τ act, -40 mV (ms)	τ deact, -80 mV (ms)	Normalized tail current $V_{0.5}$ (mV)	Non-normalized tail current $V_{0.5}$ (mV)	Slope (mV)
**Ctrl**	691 ± 54 (n =9)	222 ± 24 (n =9)	-42.8 ± 0.5 (n =9)	-41.7 ± 4.8 (n =9)	7.3 ± 0.4 (n =9)
**0.1 μM MTX**	684 ± 55 (n =5)	236 ± 12 (n =5)	-47.8 ± 0.5 (n =5) **	-47.3 ± 2.4 (n =5)	7.5 ± 0.4 (n =5)
**1 μM MTX**	611 ± 46 (n =9)	227 ± 20 (n =9)	-47.0 ± 0.9 (n =9) **	-44.4 ± 4.9 (n =9)	7.4 ± 0.6 (n =9)
**3 μM MTX**	530 ± 37 (n =9) *	259 ± 12 (n =9)	-50.7 ± 1.0 (n =9) ***	-47.3 ± 5.0 (n =9)	7.9 ± 0.8 (n =9)
**10 μM MTX**	462 ± 34 (n =9) **	261 ± 7 (n =9)	-56.2 ± 1.3 (n =9) ****	-51.9 ± 4.8 (n =9)	6.8 ± 1.3 (n =9)
**30 μM MTX**	403 ± 33 (n =9) ***	259 ± 18 (n =9)	-60.0 ± 1.8 (n =9) ****	-54.9 ± 4.9 (n =9)	8.0 ± 1.8 (n =9)
**100 μM MTX**	403 ± 34 (n =5) ***	415 ± 87 (n =5)	-57.1 ± 0.6 (n =5) ****	-56.6 ± 3.8 (n =5)	7.2 ± 0.5 (n =5)

Supplementary Table 5. Summary of Effects of MTX on KCNQ2/3 channels.

Statistics versus same channel in absence of MTX: ****p<0.0001, ***p=0.0002, **p=0.001, *p=0.02. Values indicate mean ± SEM.

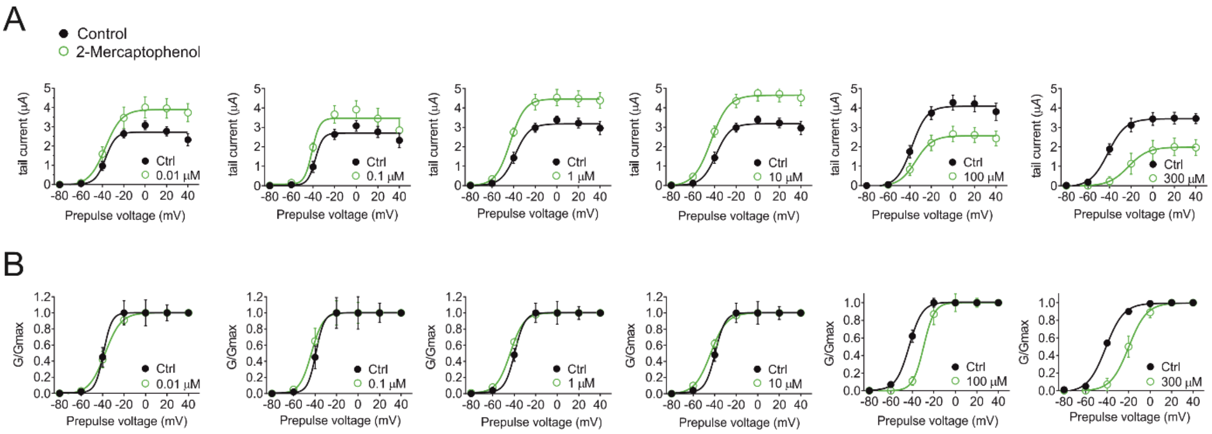

Supplementary Figure 4. Effects of 2-Mercaptophenol on KCNQ2/3 channels

A. Mean tail current versus prepulse voltage relationship for KCNQ2/3 channels in the absence (black) and presence (green) of 2-Mercaptophenol, *n* = 4-6.
B. Normalized tail current versus prepulse voltage relationships as in panel A, *n* = 4-6.
C. Dose response of KCNQ2/3 channels between -40 and +40 mV, *n* = 4-6. Error bars indicate SEM.

**KCNQ2/3**	**Normalized tail current $V_{0.5}$ (mV)**	**Non-normalized tail current $V_{0.5}$ (mV)**	**Slope (mV)**
**Ctrl**	-39.2 ± 2.8 (n =6)	-37.1 ± 1.2 (n =6)	3.8 ± 1.2 (n =6)
**0.01 μM 2-Mercaptophenol**	-37.7 ± 1.5 (n =6)	-37.2 ± 3.7 (n =6)	7.4 ± 1.6 (n =6)
**0.1 μM 2-Mercaptophenol**	-43.4 ± 3.6 (n =6)	-40.9 ± 3.2 (n =6)	5.4 ± 1.2 (n =6)
**1 μM 2-Mercaptophenol**	-43.6 ± 0.8 (n =6)	-43.2 ± 2.6 (n =6)	6.4 ± 0.9 (n =6)
**10 μM 2-Mercaptophenol**	-43.7 ± 1.1 (n =6)	-43.2 ± 2.8 (n =6)	7.4 ± 1.1 (n =6)
**100 μM 2-Mercaptophenol**	-29.5 ± 2.5 (n =6) *	-34.5 ± 4.6 (n =6)	5.0 ± 1.2 (n =6)
**300 μM 2-Mercaptophenol**	-19.8 ± 1.4 (n =4) ***	-22.5 ± 7.2 (n =4)	8.3 ± 0.9 (n =4)

Supplementary Table 6. Summary of Effects of 2-Mercaptophenol on KCNQ2/3 channels.

Statistics versus same channel in absence of 2-Mercaptophenol: *** p=0.004, * p=0.02. Values indicate mean ± SEM.

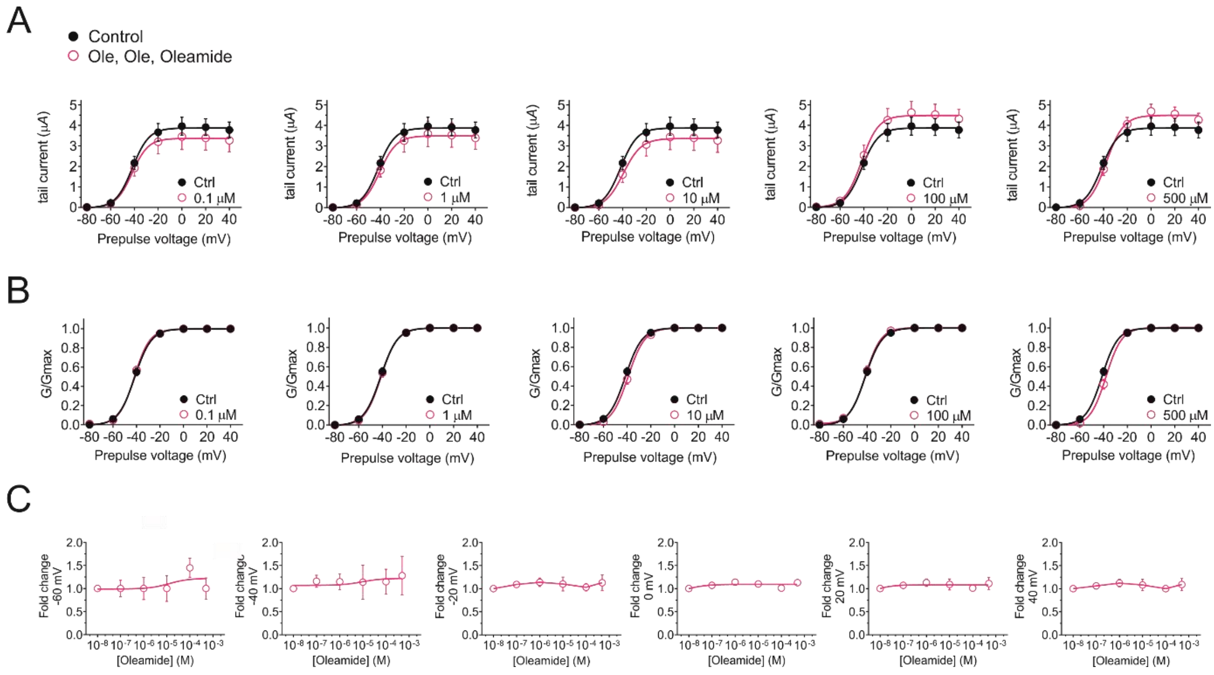

Supplementary Figure 5. Effects of Oleamide on KCNQ2/3 channels

A. Mean tail current versus prepulse voltage relationship for KCNQ2/3 channels in the absence (black) and presence (pink) of Oleamide, *n* = 8-16.

B. Normalized tail current versus prepulse voltage relationships as in panel A, *n* = 8-16.

C. Dose response of KCNQ2/3 channels between -40 and +40 mV, *n* = 8-16. Error bars indicate SEM.

KCNQ2/3	Normalized tail current $V_{0.5}$ (mV)	Non-normalized tail current $V_{0.5}$ (mV)	Slope (mV)
**Ctrl**	-41.5 ± 0.6 (n =16)	-41.7 ± 3.1 (n =16)	7.1 ± 0.8 (n =16)
**0.1 µM Oleamide**	-41.8 ± 0.6 (n =8)	-41.9 ± 4.8 (n =8)	6.5 ± 0.9 (n =8)
**1 µM Oleamide**	-41.5 ± 0.8 (n =8)	-41.6 ± 4.3 (n =8)	6.7 ± 1.1 (n =8)
**10 µM Oleamide**	-39.2 ± 0.8 (n =8)	-39.4 ± 4.8 (n =8)	7.0 ± 0.8 (n =8)
**100 µM Oleamide**	-41.5 ± 0.8 (n =9)	-41.9 ± 3.5 (n =9)	6.6 ± 0.8 (n =9)
**500 µM Oleamide**	-37.9 ± 0.8 (n =8)	-37.6 ± 2.1 (n =8)	6.7 ± 0.6 (n =8)

Supplementary Table 7. Summary of Effects of Oleamide on KCNQ2/3 channels.

Values indicate mean ± SEM.

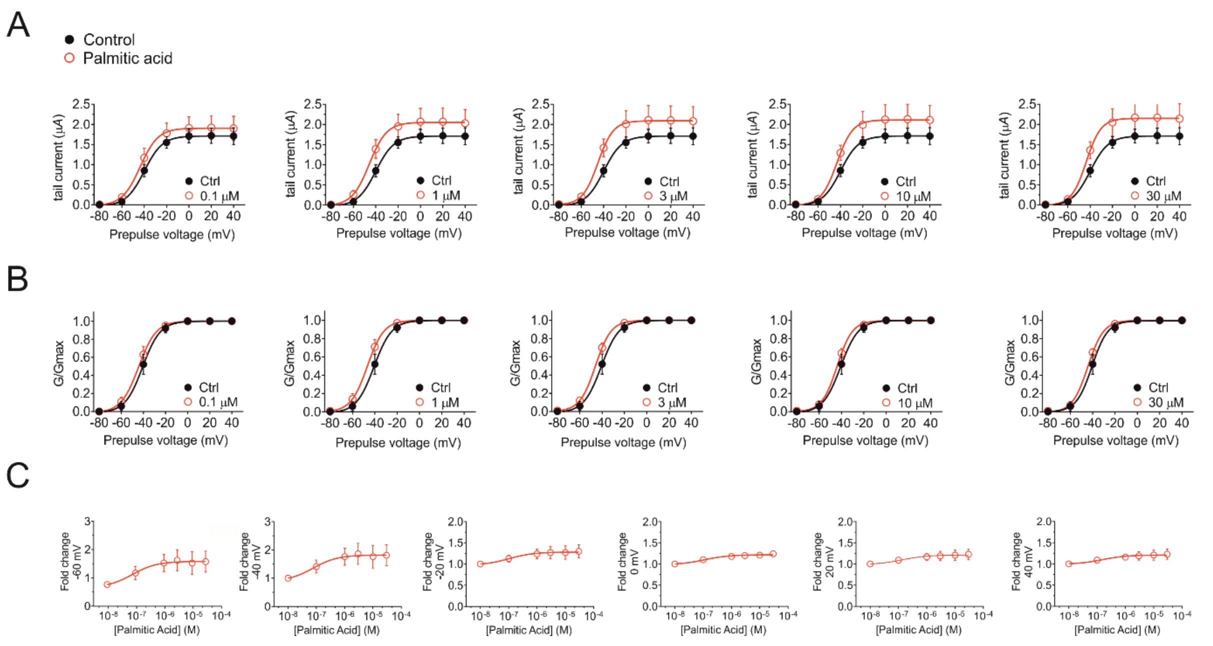

Supplementary Figure 6. Effects of palmitic acid on KCNQ2/3 channels

A. Mean tail current versus prepulse voltage relationship for KCNQ2/3 channels in the absence (black) and presence (red) of palmitic acid, *n* = 5.

B. Normalized tail current versus prepulse voltage relationships as in panel A, *n* = 5.

C. Dose response of KCNQ2/3 channels between -40 and +40 mV, *n* = 5. Error bars indicate SEM.

**KCNQ2/3**	**Normalized tail current $V_{0.5}$ (mV)**	**Non-normalized tail current $V_{0.5}$ (mV)**	**Slope (mV)**
**Ctrl**	-40.7 ± 1.9 (n =5)	-39.9 ± 3.1 (n =5)	7.9 ± 2.1 (n =5)
**0.1 µM Palmitic Acid**	-44.3 ± 1.7 (n =5)	-43.6 ± 5.0 (n =5)	7.9 ± 1.7 (n =5)
**1 µM Palmitic Acid**	-46.6 ± 1.7 (n =5)	-45.6 ± 5.5 (n =5)	7.4 ± 1.7 (n =5)
**3 µM Palmitic Acid**	-46.5 ± 1.1 (n =5)	-45.1 ± 5.4 (n =5)	7.1 ± 1.1 (n =5)
**10 µM Palmitic Acid**	-44.9 ± 0.9 (n =5)	-43.1 ± 4.9 (n =5)	6.8 ± 0.9 (n =5)
**30 µM Palmitic Acid**	-44.3 ± 1.4 (n =5)	-43.5 ± 4.9 (n =5)	7.0 ± 1.1 (n =5)

Supplementary Table 8. Summary of Effects of Palmitic acid on KCNQ2/3 channels.

Statistics versus same channel in absence of Palmitic acid: **p=0.007, *p=0.01. Values indicate mean ± SEM.

KCNQ2/3	Normalized tail current $V_{0.5}$ (mV)	Non-normalized tail current $V_{0.5}$ (mV)	Slope (mV)
**Ctrl**	-37.5 ± 1.5 (n =5)	-38.2 ± 5.8 (n =5)	6.7 ± 1.9 (n =5)
**100 µM Sorbic Acid**	-39.9 ± 0.9 (n =5)	-39.9 ± 3.0 (n =5)	4.2 ± 0.9 (n =5)

Supplementary Table 9. Summary of Effects of Sorbic acid on KCNQ2/3 channels.

Values indicate mean ± SEM.

KCNQ2/3	τ act, -60 mV (ms)	τ act, -40 mV (ms)	τ act, -20 mV (ms)
**Ctrl**	1726 ± 200 (n =9)	802 ± 69 (n =9)	276 ± 24 (n =9)
**30 µM MTX**	835 ± 51 (n =9) **	397 ± 26 (n =9) **	205 ± 6 (n =9) **

Supplementary Table 10. Summary of effects of MTX on KCNQ2/3 channel activation kinetics.

Statistics versus same channel in absence of 30 µM MTX: **p=0.002.

KCNQ2/3	τ deact, -120 mV (ms)	τ deact, -110 mV (ms)	τ deact, -100 mV (ms)	τ deact, -90 mV (ms)	τ deact, -80 mV (ms)
**Ctrl**	21 ± 1.0 (n =5)	30 ± 1.3 (n =5)	42 ± 1.9 (n =5)	58 ± 2.7 (n =5)	81 ± 26 (n =5)
**30 µM MTX**	36 ± 1.2 (n =5)	53 ± 3.5 (n =5)	77 ± 6.2 (n =5)	105 ± 4.2 (n =5)	143 ± 39 (n =5)

Supplementary Table 11. Summary of effects of MTX on KCNQ2/3 channel deactivation kinetics.

Statistics versus same channel in absence of 30 µM MTX: ****p<0.0001, ***p=0.0003, **p=0.002.

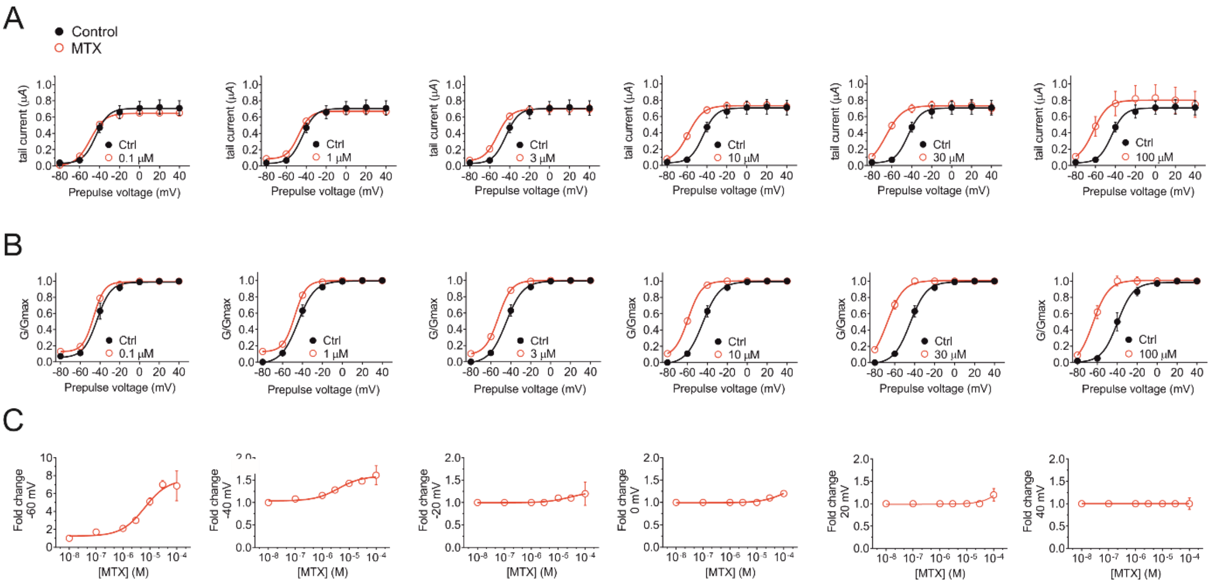

Supplementary Figure 7. Effects of MTX on KCNQ2 channels

A. Mean tail current versus prepulse voltage relationship for KCNQ2 channels in the absence (black) and presence (red) of MTX, *n* = 5-10.

B. Normalized tail current versus prepulse voltage relationships as in panel A, *n* = 5-10.

C. Dose response of KCNQ2 channels between -40 and +40 mV, *n* = 5-10. Error bars indicate SEM.

KCNQ2	τ act, -40 mV (ms)	τ deact, -80 mV (ms)	Normalized tail current $V_{0.5}$ (mV)	Non-normalized tail current $V_{0.5}$ (mV)	Slope (mV)
**Ctrl**	647 ± 47 (n =10)	390 ± 53 (n =10)	-44.5 ± 1.4 (n =10)	-44.0 ± 3.9 (n =10)	7.5 ± 0.31 (n =10)
**0.1 µM MTX**	538 ± 20 (n =5)	294 ± 10 (n =5)	-50.0 ± 0.35 (n =5) **	-50.0 ± 0.4 (n =5)	7.4 ± 0.23 (n =5)
**1 µM MTX**	490 ± 23 (n =5) *	271 ± 11 (n =5)	-51.4 ± 0.46 (n =5) ***	-51.7 ± 2.0 (n =5)	7.4 ± 0.31 (n =5)
**3 µM MTX**	417 ± 25 (n =5) ***	237 ± 12 (n =5) *	-54.9 ± 0.41 (n =5) ****	-52.1 ± 2.5 (n =5)	7.3 ± 0.33 (n =5)
**10 µM MTX**	341 ± 22 (n =5) ****	199 ± 11 (n =5) **	-61.2 ± 0.87 (n =5) ****	-61.7 ± 2.7 (n =5) **	7.0 ± 1.0 (n =5)
**30 µM MTX**	301 ± 19 (n =5) ****	173 ± 10 (n =5) **	-68.5 ± 1.5 (n =5) ****	-68.7 ± 2.7 (n =5) ***	7.4 ± 0.6 (n =5)
**100 µM MTX**	353 ± 22 (n =5) ***	190 ± 8 (n =5) **	-66.0 ± 2.7 (n =5) ****	-66.2 ± 2.7 (n =5) ***	8.1 ± 1.2 (n =5)

Supplementary Table 12. Summary of Effects of MTX on KCNQ2 channels.

Statistics versus same channel in absence of MTX: ****p<0.0001, ***p=0.0009, **p=0.005, *p=0.01. Values indicate mean ± SEM.

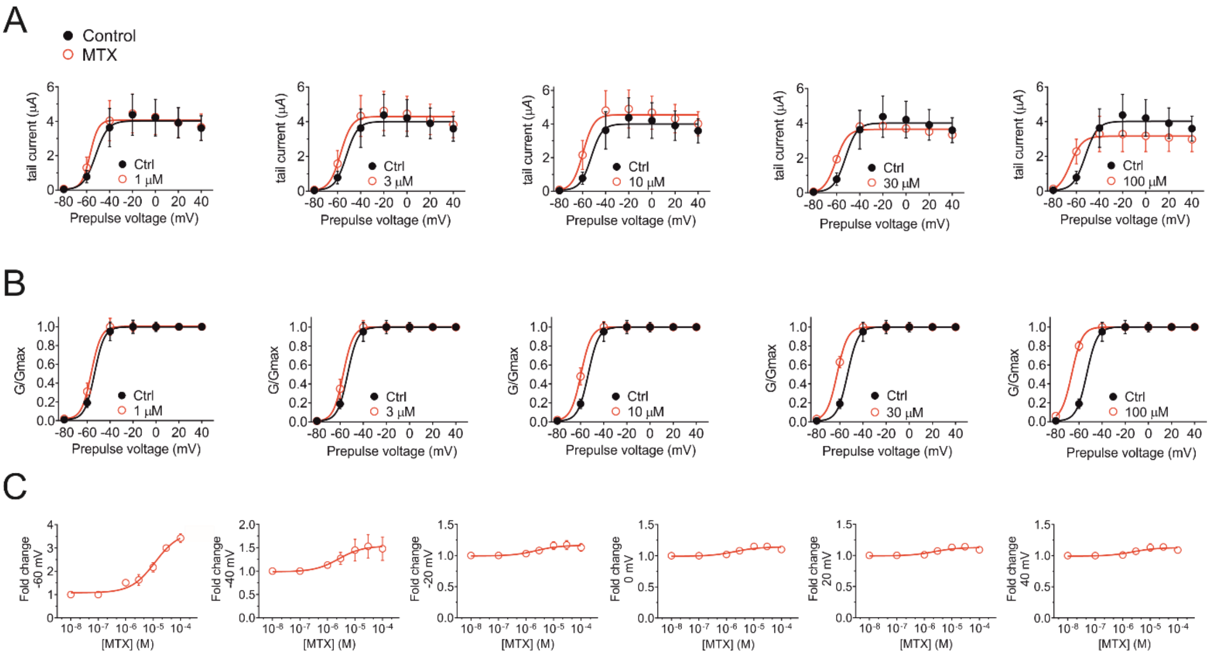

Supplementary Figure 8. Effects of MTX on KCNQ3* channels

A. Mean tail current versus prepulse voltage relationship for KCNQ3* channels in the absence (black) and presence (red) of MTX, *n* = 5.

B. Normalized tail current versus prepulse voltage relationships as in panel A, *n* = 5.

C. Dose response of KCNQ3* channels between -40 and +40 mV, *n* = 5. Error bars indicate SEM.

**KCNQ3***	**τ act, -40 mV (ms)**	**τ deact, -80 mV (ms)**	**Normalized tail current $V_{0.5}$ (mV)**	**Non-normalized tail current $V_{0.5}$ (mV)**	**Slope (mV)**
**Ctrl**	686 ± 124 (n =5)	373 ± 70 (n =5)	-53.6 ± 2.3 (n =5)	-53.1 ± 8.5 (n =5)	n.d.
**1 µM MTX**	552 ± 98 (n =5)	290 ± 51 (n =5)	-56.4 ± 1.6 (n =5)	-56.1 ± 4.2 (n =5)	n.d.
**3 µM MTX**	489 ± 55 (n =5)	263 ± 29 (n =5)	-57.3 ± 1.4 (n =5)	-57.4 ± 5.3 (n =5)	n.d.
**10 µM MTX**	421 ± 26 (n =5)	244 ± 17 (n =5)	-59.5 ± 1.0 (n =5)	-59.5 ± 4.2 (n =5)	n.d.
**30 µM MTX**	388 ± 20 (n =5)	240 ± 18 (n =5)	-62.3 ± 1.2 (n =5) *	-62.1 ± 3.9 (n =5)	n.d.
**100 µM MTX**	403 ± 36 (n =5)	269 ± 30 (n =5)	-66.2 ± 2.1 (n =5) **	-65.2 ± 3.9 (n =5)	n.d.

Supplementary Table 13. Summary of Effects of MTX on KCNQ3* channels.

Statistics versus same channel in absence of MTX: **p=0.005, *p=0.01. Values indicate mean ± SEM. n.d., not determined.

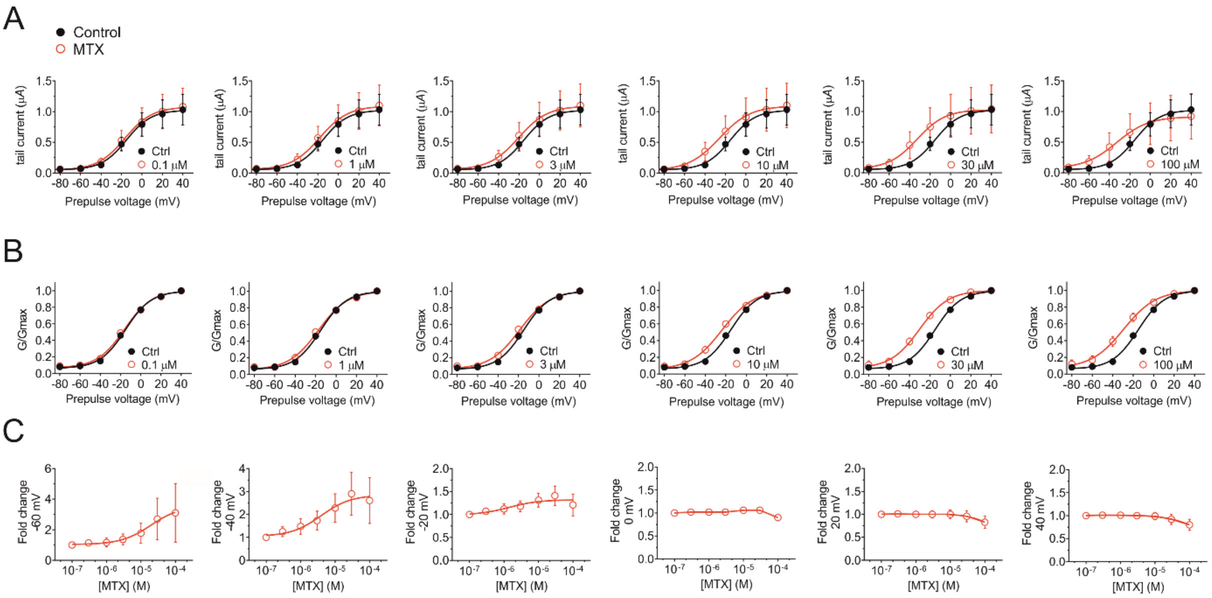

Supplementary Figure 9. Effects of MTX on KCNQ4 channels

A. Mean tail current versus prepulse voltage relationship for KCNQ4 channels in the absence (black) and presence (red) of MTX, *n* = 5.

B. Normalized tail current versus prepulse voltage relationships as in panel A, *n* = 5.

C. Dose response of KCNQ4 channels between -40 and +40 mV, *n* = 5. Error bars indicate SEM.

KCNQ4	τ act, -40 mV (ms)	τ deact, -80 mV (ms)	Normalized tail current $V_{0.5}$ (mV)	Non-normalized tail current $V_{0.5}$ (mV)	Slope (mV)
**Ctrl**	1015 ± 75 (n =5)	1185 ± 258 (n =5)	-15.5 ± 1.3 (n =5)	-15.4 ± 6.5 (n =5)	12.6 ± 1.2 (n =5)
**0.1 μM MTX**	941 ± 52 (n =5)	924 ± 223 (n =5)	-17.0 ± 1.6 (n =5)	-17.1 ± 8.1 (n =5)	14.1 ± 1.6 (n =5)
**1 μM MTX**	878 ± 78 (n =5)	891 ± 234 (n =5)	-18.1 ± 1.9 (n =5)	-18.1 ± 9.0 (n =5)	15.0 ± 1.9 (n =5)
**3 μM MTX**	851 ± 70 (n =5)	861 ± 240 (n =5)	-20.1 ± 1.3 (n =5) *	-20.5 ± 9.8 (n =5)	15.6 ± 1.9 (n =5)
**10 μM MTX**	847 ± 94 (n =5)	906 ± 293 (n =5)	-25.6 ± 1.9 (n =5) **	-25.3 ± 9.7 (n =5)	15.9 ± 1.9 (n =5)
**30 μM MTX**	865 ± 108 (n =5)	905 ± 283 (n =5)	-32.1 ± 1.9 (n =5) ***	-32.9 ± 9.7 (n =5)	14.4 ± 1.8 (n =5)
**100 μM MTX**	1092 ± 170 (n =5)	1197 ± 446 (n =5)	-31.5 ± 1.3 (n =5) ****	-31.3 ± 9.4 (n =5)	16.4 ± 3.2 (n =5)

Supplementary Table 14. Summary of Effects of MTX on KCNQ4 channels.

Statistics versus same channel in absence of MTX: ****p<0.0001, ***p=0.0009, **p=0.005, *p=0.01. Values indicate mean ± SEM.

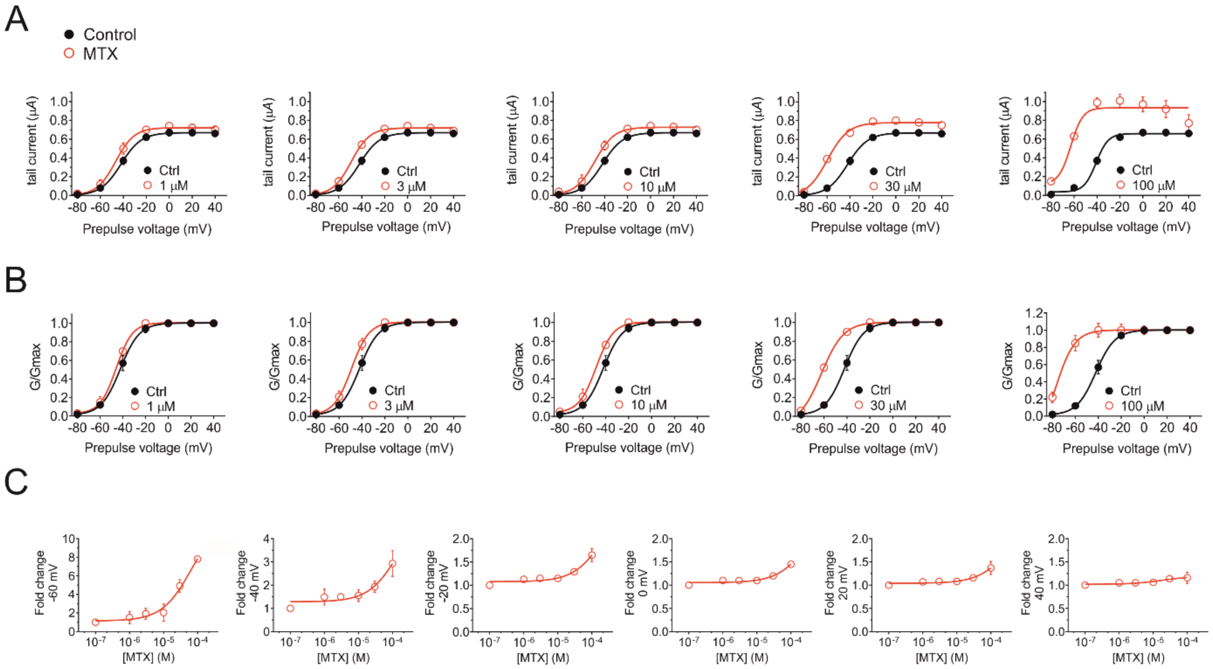

Supplementary Figure 10. Effects of MTX on KCNQ5 channels

A. Mean tail current versus prepulse voltage relationship for KCNQ5 channels in the absence (black) and presence (red) of MTX, *n* = 5.

B. Normalized tail current versus prepulse voltage relationships as in panel A, *n* = 5.

C. Dose response of KCNQ5 channels between -40 and +40 mV, *n* = 5. Error bars indicate SEM.

**KCNQ5**	**Normalized tail current $V_{0.5}$ (mV)**	**Non-normalized tail current $V_{0.5}$ (mV)**	**Slope (mV)**
**Ctrl**	-42.4 ± 1.6 (n =5)	-41.9 ± 1.0 (n =5)	7.2 ± 0.8 (n =5)
**1 μM MTX**	-49.9 ± 2.1 (n =5) *	-49.3 ± 1.2 (n =5) **	8.3 ± 2.1 (n =5)
**3 μM MTX**	-51.1 ± 2.3 (n =5) *	-51.7 ± 1.8 (n =5) **	8.5 ± 2.1 (n =5)
**10 μM MTX**	-51.5 ± 3.2 (n =5) *	-51.9 ± 0.8 (n =5) ****	8.6 ± 2.9 (n =5)
**30 μM MTX**	-55.6 ± 3.8 (n =5) *	-55.9 ± 0.8 (n =5) ****	8.8 ± 3.6 (n =5)
**100 μM MTX**	-71.6 ± 2.1 (n =5) ****	-71.1 ± 2.6 (n =5) ***	n.d.

Supplementary Table 15. Summary of Effects of MTX on KCNQ5 channels.

Statistics versus same channel in absence of MTX: ****p<0.0001, **p=0.005, *p=0.01. Values indicate mean ± SEM. n.d., not determined.

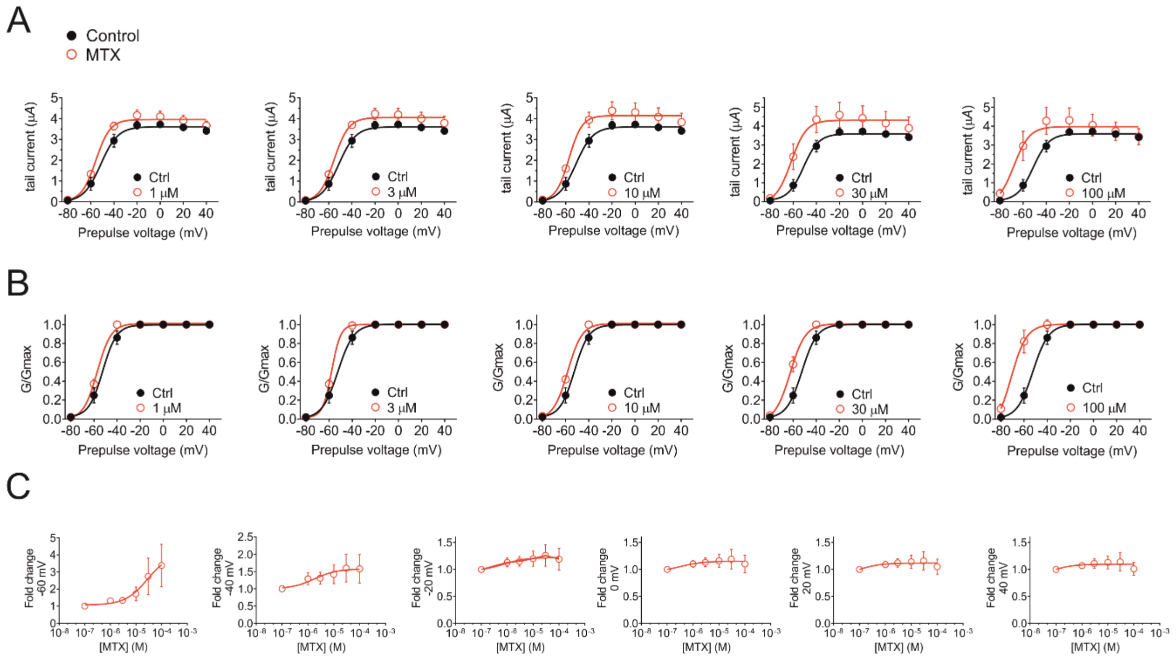

Supplementary Figure 11. Effects of MTX on W236L-Q2/Q3 channels

A. Mean tail current versus prepulse voltage relationship for W236L-Q2/Q3 channels in the absence (black) and presence (red) of MTX, *n* = 3.

B. Normalized tail current versus prepulse voltage relationships as in panel A, *n* = 3.

C. Dose response of W236L-Q2/Q3 channels between -40 and +40 mV, *n* = 3. Error bars indicate SEM.

W236L-Q2/Q3	Normalized tail current $V_{0.5}$ (mV)	Non-normalized tail current $V_{0.5}$ (mV)	Slope (mV)
**Ctrl**	-52.4 ± 1.3 (n =3)	-51.2 ± 2.5 (n =3)	6.0 ± 0.7 (n =3)
**1 µM MTX**	-57.1 ± 0.9 (n =3)	-55.7 ± 1.9 (n =3)	6.0 ± 0.7 (n =3)
**3 µM MTX**	-57.8 ± 1.3 (n =3)	-55.4 ± 2.1 (n =3)	3.9 ± 2.2 (n =3)
**10 µM MTX**	-58.1 ± 0.9 (n =3)	-57.3 ± 2.7 (n =3)	6.2 ± 0.7 (n =3)
**30 µM MTX**	-62.4 ± 1.3 (n =3)	-61.9 ± 3.9 (n =3)	6.6 ± 0.9 (n =3)
**100 µM MTX**	-71.4 ± 4.5 (n =3)	-67.9 ± 7.7 (n =3)	6.8 ± 1.2 (n =3)

Supplementary Table 16. Summary of Effects of MTX on W236L-Q2/Q3 channels.

Statistics versus same channel in absence of MTX: **p=0.004, *p=0.03. Values indicate mean ± SEM.

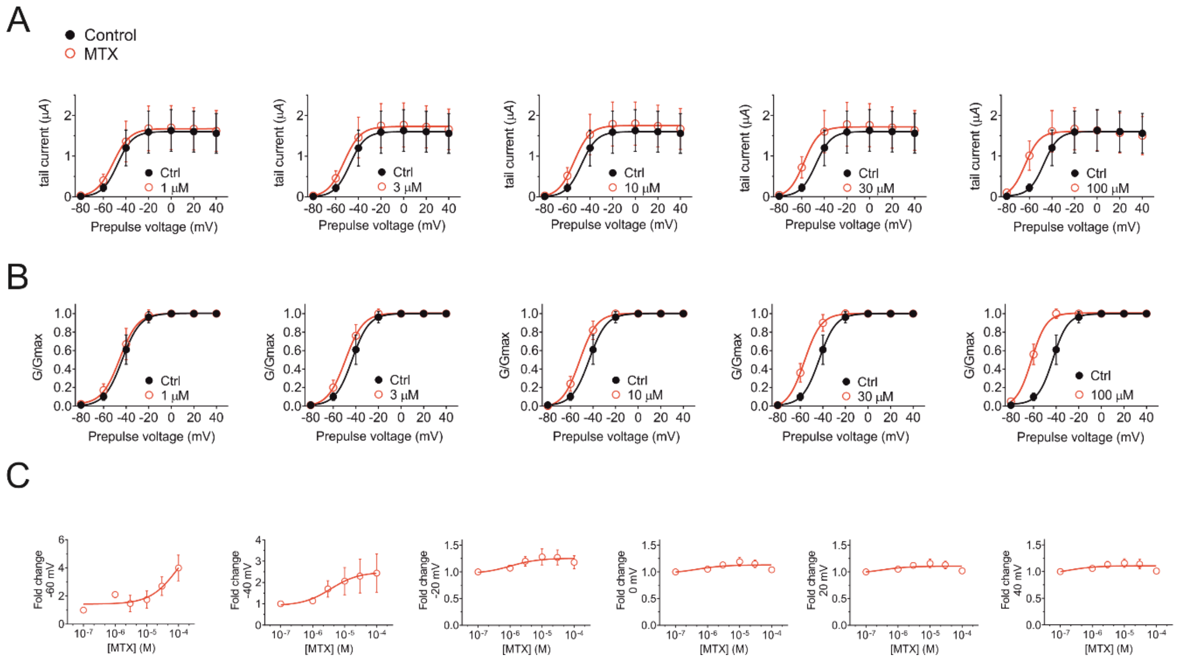

Supplementary Figure 12. Effects of MTX on Q2/W265L-Q3 channels

A. Mean tail current versus prepulse voltage relationship for Q2/W265L-Q3 channels in the absence (black) and presence (red) of MTX, *n* = 3.

B. Normalized tail current versus prepulse voltage relationships as in panel A, *n* = 3.

C. Dose response of Q2/W265L-Q3 channels between -40 and +40 mV, *n* = 3. Error bars indicate SEM.

Q2/W265L-Q3	Normalized tail current $V_{0.5}$ (mV)	Non-normalized tail current $V_{0.5}$ (mV)	Slope (mV)
**Ctrl**	-43.5 ± 2.7 (n =3)	-47.5 ± 10.9 (n =3)	7.9 ± 1.7 (n =3)
**1 μM MTX**	-45.7 ± 2.9 (n =3)	-51.3 ± 11.3 (n =3)	7.9 ± 1.7 (n =3)
**3 μM MTX**	-49.2 ± 2.6 (n =3)	-52.7 ± 10.5 (n =3)	7.9 ± 1.8 (n =3)
**10 μM MTX**	-51.8 ± 2.4 (n =3)	-54.0 ± 9.7 (n =3)	7.6 ± 1.4 (n =3)
**30 μM MTX**	-56.3 ± 2.2 (n =3)	-58.0 ± 8.3 (n =3)	7.4 ± 1.6 (n =3)
**100 μM MTX**	-61.8 ± 2.2 (n =3)	-63.7 ± 11.2 (n =3)	6.6 ± 2.1 (n =3)

Supplementary Table 17. Summary of Effects of MTX on Q2/W265L-Q3 channels.

Statistics versus same channel in absence of MTX: **p=0.004, *p=0.03. Values indicate mean ± SEM.

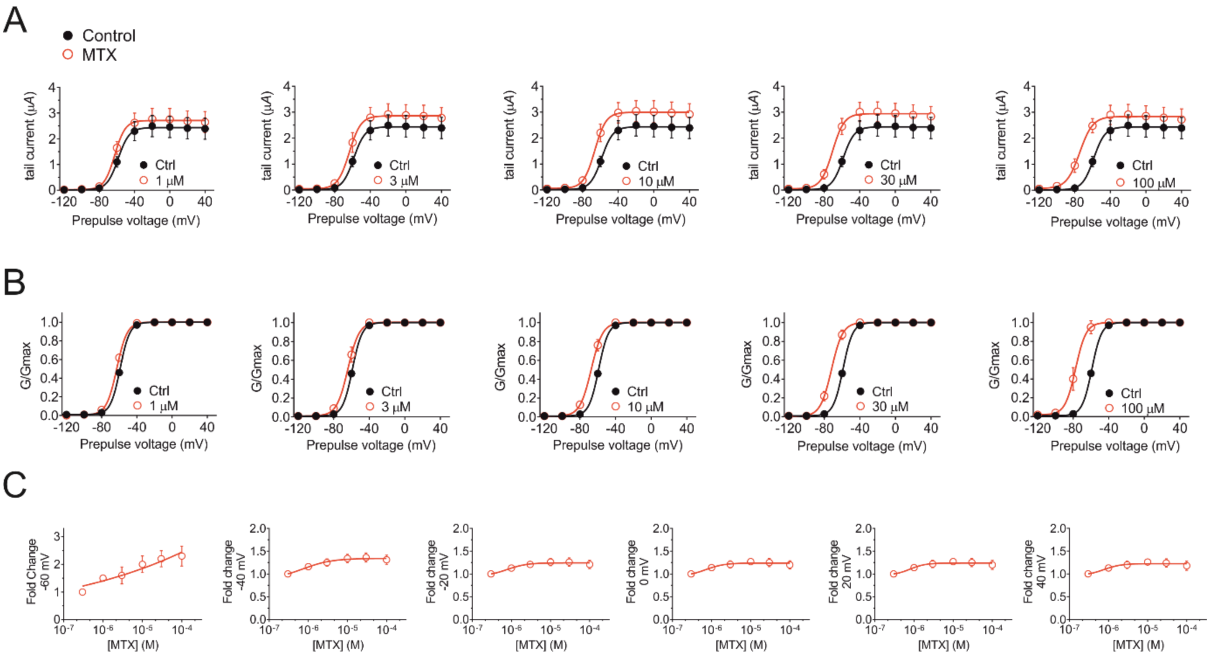

Supplementary Figure 13. Effects of MTX on W236L-Q2/W265L-Q3 channels

A. Mean tail current versus prepulse voltage relationship for W236L-Q2/W265L-Q3 channels in the absence (black) and presence (red) of MTX, *n* = 5.

B. Normalized tail current versus prepulse voltage relationships as in panel A, *n* = 5.

C. Dose response of W236L-Q2/W265L-Q3 channels between -40 and +40 mV, *n* = 5. Error bars indicate SEM.

**W236L-Q2/ W265L-Q3**	**Normalized tail current $V_{0.5}$ (mV)**	**Non-normalized tail current $V_{0.5}$ (mV)**	**Slope (mV)**
**Ctrl**	-59.0 ± 0.2 (n =5)	-58.7 ± 3.4 (n =5)	5.5 ± 0.4 (n =5)
**1 μM MTX**	-62.8 ± 0.4 (n =5)	-62.5 ± 3.4 (n =5)	5.9 ± 0.5 (n =5)
**3 μM MTX**	-64.1 ± 0.9 (n =5)	-63.6 ± 3.7 (n =5)	6.3 ± 0.9 (n =5)
**10 μM MTX**	-67.3 ± 0.9 (n =5)	-66.3 ± 3.9 (n =5)	6.4 ± 0.6 (n =5)
**30 μM MTX**	-71.9 ± 0.9 (n =5)	-70.6 ± 3.7 (n =5)	6.3 ± 0.5 (n =5)
**100 μM MTX**	-77.3 ± 1.4 (n =5)	-74.4 ± 3.9 (n =5)	5.9 ± 1.8 (n =5)

Supplementary Table 18. Summary of Effects of MTX on KCNQ2-W236L/KCNQ3-W265L channels.

Statistics versus same channel in absence of MTX: **p=0.004, *p=0.03. Values indicate mean ± SEM.

KCNQ2/3	Normalized tail current $V_{0.5}$ (mV)	Non-normalized tail current $V_{0.5}$ (mV)
**Ctrl**	-47.7 ± 1.6 (n =6)	-47.8 ± 4.0 (n =6)
**100 µM GABOB 30 µM MTX**	-59.7 ± 1.8 (n =6) ***	-60.8 ± 4.5 (n =6)

Supplementary Table 19. Summary of effects of GABOB on MTX sensitivity of KCNQ2/3 channels.

Statistics versus same channel in absence of 100 µM GABOB and 30 µM MTX: ***p=0.0007.

W236L-Q2/ W265L-Q3	Normalized tail current $V_{0.5}$ (mV)	Non-normalized tail current $V_{0.5}$ (mV)
**Ctrl**	-51.9 ± 3.2 (n =9)	-53.4 ± 0.8 (n =9)
**100 µM 2-MP**	-51.0 ± 4.2 (n =9)	-53.8 ± 1.7 (n =9)

Supplementary Table 20. Summary of effects of 2-mercaptophenol on KCNQ2-W236L/KCNQ3-W265L channels.

KCNQ2/3	Normalized tail current $V_{0.5}$ (mV)	Non-normalized tail current $V_{0.5}$ (mV)
**Ctrl**	-40.5 ± 0.7 (n =9)	-41.1 ± 3.0 (n =9)
**100 µM 2-MP 30 µM MTX**	-51.8 ± 1.7 (n =9) ****	-52.0 ± 4.7 (n =9) ****

Supplementary Table 21. Summary of effects of 2-mercaptophenol on MTX sensitivity of KCNQ2/3 channels.

Statistics versus same channel in absence of 100 µM 2-mercaptophenol and 30 µM MTX: ****p<0.0001.

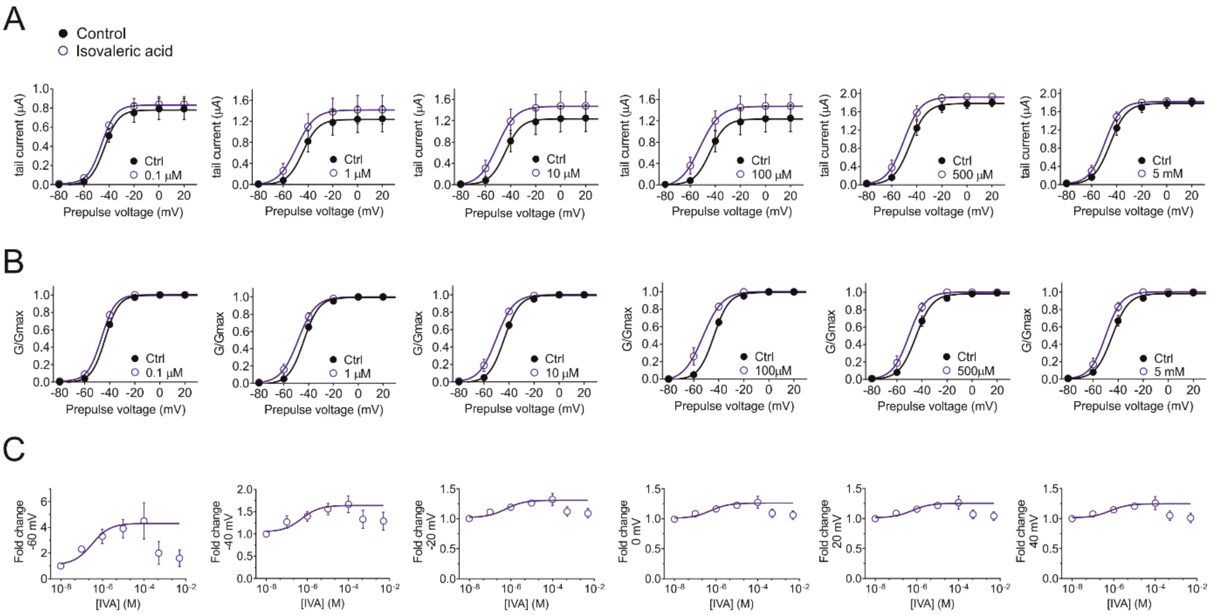

Supplementary Figure 14. Effects of Isovaleric acid on KCNQ2 channels

A. Mean tail current versus prepulse voltage relationship for KCNQ2 channels in the absence (black) and presence (blue) of Isovaleric acid, *n* = 4-6.
B. Normalized tail current versus prepulse voltage relationships as in panel A, *n* = 4-6.
C. Dose response of KCNQ2 channels between -40 and +40 mV, *n* = 4-6. Error bars indicate SEM.

**KCNQ2**	**Normalized tail current $V_{0.5}$ (mV)**	**Non-normalized tail current $V_{0.5}$ (mV)**	**Slope (mV)**
**Ctrl**	-43.7 ± 0.6 (n =6)	-43.6 ± 4.0 (n =6)	5.5 ± 0.6 (n =6)
**0.1 μM IVA**	-46.6 ± 0.9 (n =4) *	-45.9 ± 3.1 (n =4)	5.5 ± 0.9 (n =4)
**1 μM IVA**	-48.5 ± 1.3 (n =4) *	-49.3 ± 6.9 (n =4)	7.0 ± 0.8 (n =4)
**10 μM IVA**	-50.1 ± 1.2 (n =4) **	-50.7 ± 6.5 (n =4)	6.9 ± 0.8 (n =4)
**100 μM IVA**	-52.6 ± 1.8 (n =4) *	-52.2 ± 5.6 (n =4)	7.8 ± 1.8 (n =4)
**500 μM IVA**	-50.4 ± 1.5 (n =4) *	-50.4 ± 2.1 (n =4)	6.6 ± 0.9 (n =4)
**5 mM IVA**	-49.9 ± 1.4 (n =4) *	-49.5 ± 1.4 (n =4)	6.3 ± 0.8 (n =4)

Supplementary Table 22. Summary of Effects of IVA on KCNQ2 channels.

Statistics versus same channel in absence of IVA: **p=0.006, *p=0.04. Values indicate mean ± SEM.

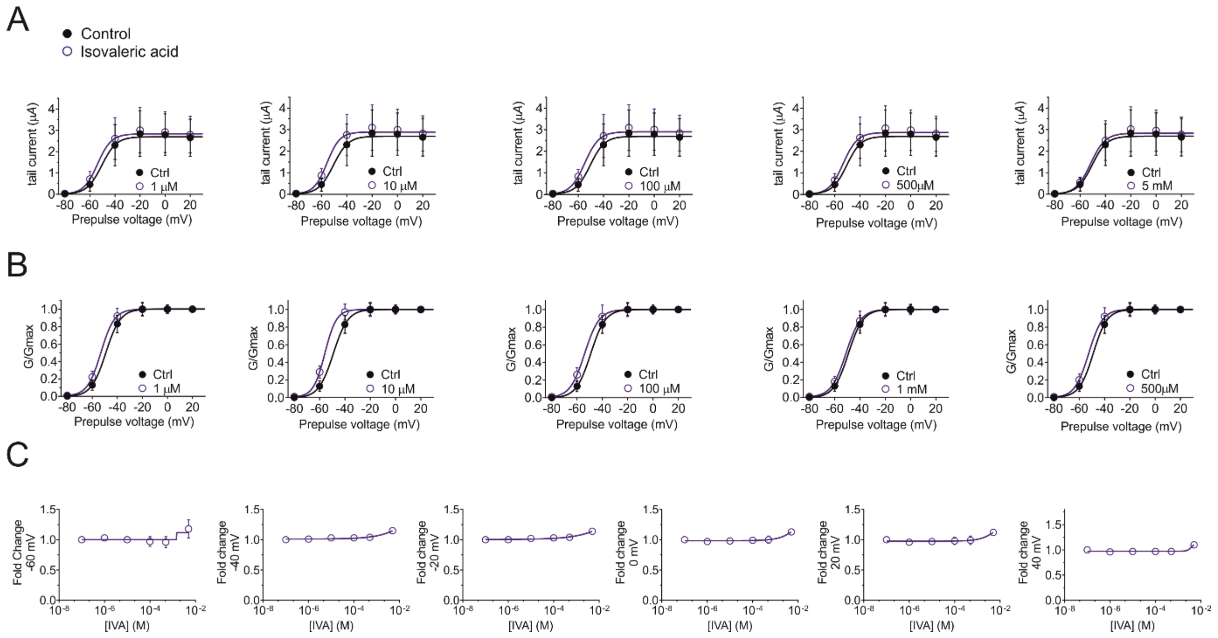

Supplementary Figure 15. Effects of Isovaleric acid on KCNQ3* channels

A. Mean tail current versus prepulse voltage relationship for KCNQ3* channels in the absence (black) and presence (blue) of Isovaleric acid, *n* = 4.

B. Normalized tail current versus prepulse voltage relationships as in panel A, *n* = 4.

C. Dose response of KCNQ3* channels between -40 and +40 mV, *n* = 4. Error bars indicate SEM.

KCNQ3*	Normalized tail current $V_{0.5}$ (mV)	Non-normalized tail current $V_{0.5}$ (mV)	Slope (mV)
**Ctrl**	-49.2 ± 2.2 (n =4)	-50.6 ± 1.6 (n =4)	n.d.
**1 µM IVA**	-53.1 ± 2.1 (n =4)	-53.9 ± 1.3 (n =4)	n.d.
**10 µM IVA**	-55.8 ± 2.2 (n =4)	-55.6 ± 1.5 (n =4)	n.d.
**100 µM IVA**	-53.9 ± 2.2 (n =4)	-54.8 ± 0.9 (n =4)	n.d.
**500 µM IVA**	-52.6 ± 2.1 (n =4)	-53.3 ±1.7 (n =4)	n.d.
**5 mM IVA**	-51.0 ± 2.3 (n =4)	-51.7 ± 1.5 (n =4)	n.d.

Supplementary Table 23. Summary of Effects of IVA on KCNQ3* channels.

Statistics versus same channel in absence of IVA: **p=0.007, *p=0.01. Values indicate mean ± SEM. n.d., not determined.

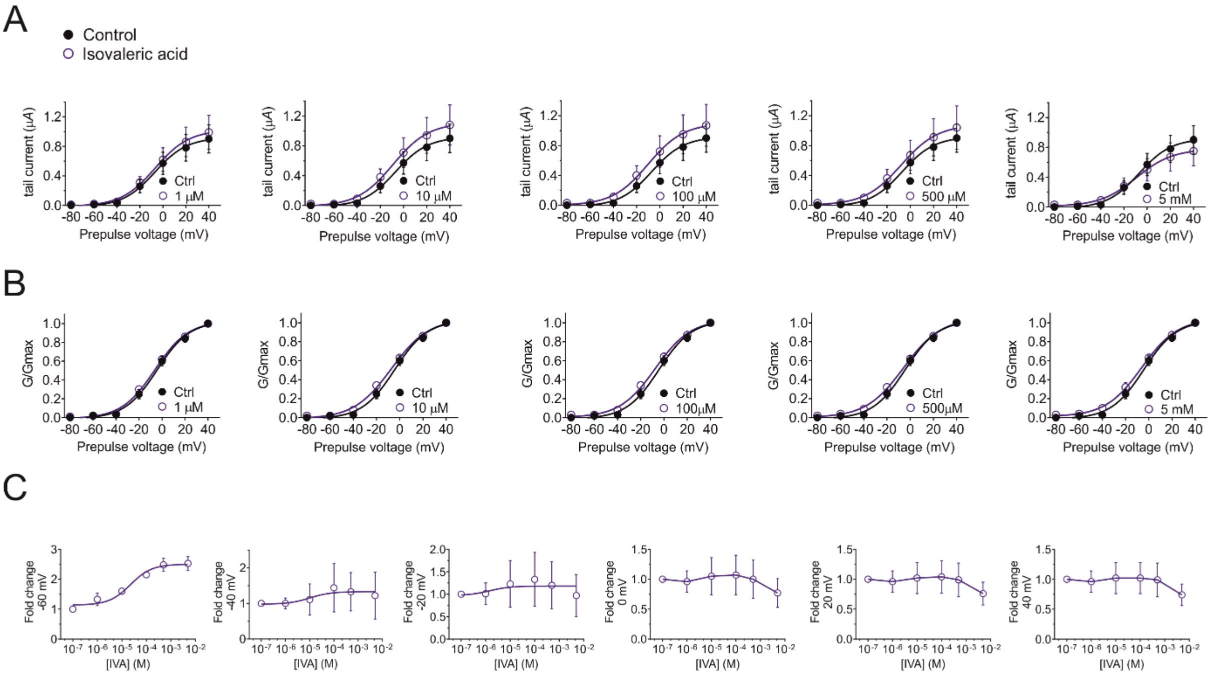

Supplementary Figure 16. Effects of Isovaleric acid on KCNQ4 channels

A. Mean tail current versus prepulse voltage relationship for KCNQ4 channels in the absence (black) and presence (blue) of Isovaleric acid, *n* = 4.

B. Normalized tail current versus prepulse voltage relationships as in panel A, *n* = 4.

C. Dose response of KCNQ4 channels between -40 and +40 mV, *n* = 4. Error bars indicate SEM.

**KCNQ4**	**Normalized tail current $V_{0.5}$ (mV)**	**Non-normalized tail current $V_{0.5}$ (mV)**	**Slope (mV)**
**Ctrl**	-4.3 ± 2.2 (n =4)	-6.9 ± 8.2 (n =4)	14.1 ± 2.0 (n =4)
**1 μM IVA**	-6.2 ± 1.4 (n =4)	-6.7 ± 9.1 (n =4)	14.6 ± 1.4 (n =4)
**10 μM IVA**	-7.2 ± 1.6 (n =4)	-9.1 ± 10.4 (n =4)	16.2 ± 1.5 (n =4)
**100 μM IVA**	-7.5 ± 1.8 (n =4)	-10.2 ± 11.1 (n =4)	15.3 ± 1.8 (n =4)
**500 μM IVA**	-7.5 ± 5.7 (n =4)	-7.8 ± 11.9 (n =4)	16.2 ± 1.6 (n =4)
**5 mM IVA**	-6.1 ± 2.1 (n =4)	-9.3 ± 12.1 (n =4)	15.6 ± 1.9 (n =4)

Supplementary Table 24. Summary of Effects of IVA on KCNQ4 channels.

Values indicate mean ± SEM.

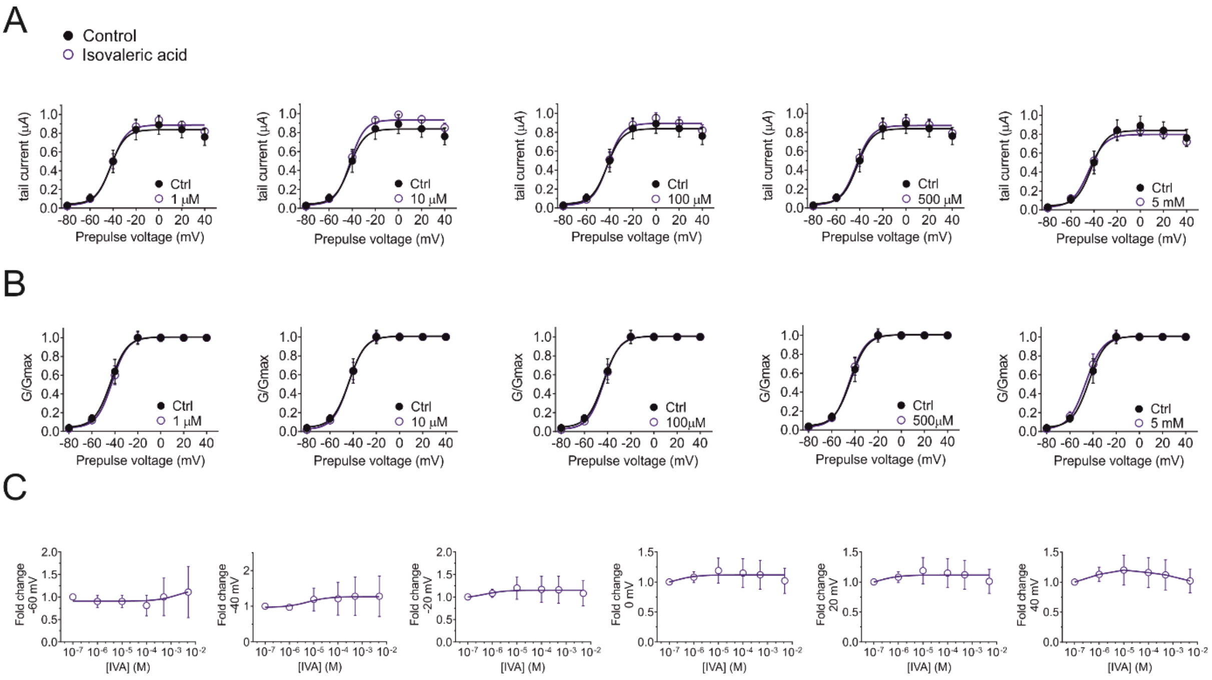

Supplementary Figure 17. Effects of Isovaleric acid on KCNQ5 channels

A. Mean tail current versus prepulse voltage relationship for KCNQ5 channels in the absence (black) and presence (blue) of Isovaleric acid, *n* = 4.

B. Normalized tail current versus prepulse voltage relationships as in panel A, *n* = 4.

C. Dose response of KCNQ5 channels between -40 and +40 mV, *n* = 4. Error bars indicate SEM.

KCNQ5	Normalized tail current $V_{0.5}$ (mV)	Non-normalized tail current $V_{0.5}$ (mV)	Slope (mV)
**Ctrl**	-43.7 ± 2.4 (n =4)	-42.2 ± 3.8 (n =4)	7.1 ± 2.4 (n =4)
**1 µM IVA**	-42.4 ± 1.7 (n =4)	-41.5 ± 1.9 (n =4)	6.8 ± 2.1 (n =4)
**10 µM IVA**	-43.8 ± 1.7 (n =4)	-41.8 ± 1.6 (n =4)	6.9 ± 1.7 (n =4)
**100 µM IVA**	-43.4 ± 1.4 (n =4)	-41.6 ± 2.1 (n =4)	6.7 ± 1.5 (n =4)
**500 µM IVA**	-44.6 ± 1.8 (n =4)	-42.9 ± 2.7 (n =4)	6.9 ± 1.6 (n =4)
**5 mM IVA**	-46.3 ± 2.1 (n =4)	-44.6 ± 3.2 (n =4)	7.3 ± 1.6 (n =4)

Supplementary Table 25. Summary of Effects of IVA on KCNQ5 channels.

Statistics versus same channel in absence of IVA: ****p<0.0001, ***p=0.0009, **p=0.005, *p=0.01. Values indicate mean ± SEM.

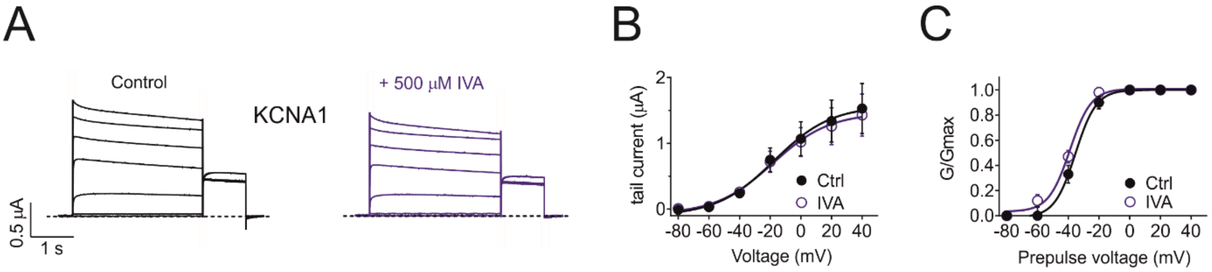

Supplementary Figure 18. Effects of Isovaleric acid on KCNA1 channels

A. Averaged KCNA1 traces in the absence (black) and presence (blue) of Isovaleric acid (500 µM), *n* = 6.
B. Mean tail current versus prepulse voltage as in panel A, *n* = 6.
C. Normalized tail current versus prepulse voltage relationships as in panel A, *n* = 6.

**KCNQ2/3**	**Normalized tail current $V_{0.5}$ (mV)**	**Non-normalized tail current $V_{0.5}$ (mV)**	**Slope (mV)**
**Ctrl**	-35.3 ± 1.5 (n =6)	-37.5 ± 2.4 (n =6)	6.8 ± 1.3 (n =6)
**500 µM IVA**	-38.9 ± 1.1 (n =6)	-37.8 ± 3.2 (n =6)	6.9 ± 1.1 (n =6)

Supplementary Table 26. Summary of Effects of Isovaleric acid on KCNA1 channels.

Values indicate mean ± SEM.

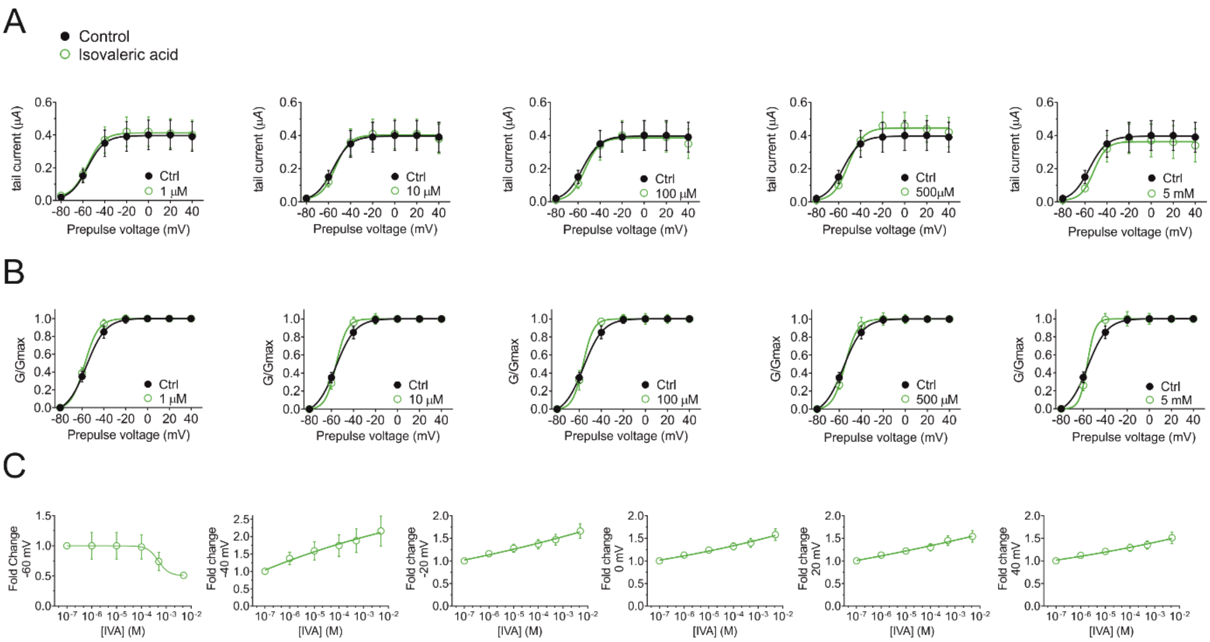

Supplementary Figure 19. Effects of Isovaleric acid on W236L-Q2/W265L-Q3 channels

A. Mean tail current versus prepulse voltage relationship for W236L-Q2/W265L-Q3 channels in the absence (black) and presence (green) of Isovaleric acid, *n* = 3-6.

B. Normalized tail current versus prepulse voltage relationships as in panel A, *n* = 3-6.

C. Dose response of W236L-Q2/W265L-Q3 channels between -40 and +40 mV, *n* = 3-6. Error bars indicate SEM.

**W236L-Q2/ W265L-Q3**	**Normalized tail current $V_{0.5}$ (mV)**	**Non-normalized tail current $V_{0.5}$ (mV)**	**Slope (mV)**
**Ctrl**	-56.0 ± 1.9 (n =6)	-55.9 ± 8.3 (n =6)	8.8 ± 1.6 (n =6)
**1 μM IVA**	-57.6 ± 1.1 (n =6)	-55.8 ± 7.5 (n =6)	6.3 ± 1.4 (n =6)
**10 μM IVA**	-55.7 ± 1.7 (n =6)	-53.7 ± 7.4 (n =6)	4.9 ± 1.6 (n =6)
**100 μM IVA**	-56.5 ± 2.0 (n =6)	-53.7 ± 7.7 (n =6)	4.7 ± 2.4 (n =6)
**500 μM IVA**	-54.6 ± 1.8 (n =4)	-51.1 ± 6.1 (n =4)	5.6 ± 1.4 (n =4)
**5 mM IVA**	-56.3 ± 3.7 (n =3)	-51.9 ± 8.4 (n =3)	3.5 ± 3.5 (n =3)

Supplementary Table 27. Summary of Effects of IVA on W236L-Q2/W265L-Q3 channels.

Values indicate mean ± SEM.

	MTX EC_{50} (µM)	IVA EC_{50} (µM)
**KCNQ2**	6.4 ± 0.21 (n =5-10)	0.34 ± 0.6 (n =4-6)
**KCNQ3***	13 ± 0.15 (n =5)	0.5 ± 1.5 (n =5)
**KCNQ4**	20.2 ± 0.9 (n =5)	16.2 ± 0.3 (n =4)
**KCNQ5**	67.1 ± 0.27 (n =5)	1939 ± 4.7 (n =4)
**KCNQ2/3**	11.5 ± 0.2 (n =5-9)	228 ± 0.4 (n =4)
**W236L-Q2/Q3**	27 ± 0.6 (n =3)	n.d.
**Q2/W265L-Q3**	70 ± 0.6 (n =3)	n.d.
**W236L-Q2/ W265L-Q3**	n.d.	n.d.

Supplementary Table 28. EC_{50} (µM) values calculated from dose responses.

Values indicate mean ± SEM. n.d., not determined.

	MTX $V_{0.5}$ EC_{50} (µM)
**KCNQ2/3**	4.0 ± 0.2 (n =5-9)
**W236L-Q2/Q3**	37.3 ± 0.2 (n =5)
**Q2/W265L-Q3**	13.7 ± 0.3 (n =5)
**W236L-Q2/ W265L-Q3**	14.9 ± 0.1 (n =6)

Supplementary Table 29. MTX EC_{50} (µM) values calculated from shift in $V_{0.5}$.

Values indicate mean ± SEM. IVA values not calculated because double mutant eliminates sensitivity.

**KCNQ2**	**Normalized tail current $V_{0.5}$ (mV)**	**Non-normalized tail current $V_{0.5}$ (mV)**
**Ctrl**	-35.7 ± 1.4 (n =5)	-33.5 ± 4.5 (n =5)
**100 µM IVA 100 µM GABOB**	-41.2 ± 1.3 (n =5) *	-39.9 ± 3.5 (n =5)

Supplementary Table 30. Summary of effects of GABOB on IVA sensitivity of KCNQ2 channels.

Statistics versus same channel in absence of 100 µM GABOB and 100 µM IVA: *p=0.05.

KCNQ2/3	Normalized tail current $V_{0.5}$ (mV)	Non-normalized tail current $V_{0.5}$ (mV)
**Ctrl**	-41.2 ± 0.6 (n =5)	-40.2 ± 2.9 (n =5)
**30 µM MTX 500 µM IVA**	-63.9 ± 1.5 (n =5) ****	-60.3 ± 2.8 (n =5) **

Supplementary Table 31. Summary of Effects of MTX and IVA on KCNQ2/3 channels.

Statistics versus same channel in absence of MTX and IVA: ****p<0.0001, **p=0.001

KCNQ2/3	τ act, -60 mV (ms)	τ act, -40 mV (ms)	τ act, -20 mV (ms)
**Ctrl**	1305 ± 149 (n =8)	665 ± 48 (n =8)	248 ± 16 (n =8)
**Cocktail**	752 ± 59 (n =8) **	384 ± 53 (n =8) **	188 ± 12 (n =8) *

Supplementary Table 32. Summary of effects of cocktail on KCNQ2/3 channel activation kinetics.

Statistics versus same channel in absence of cocktail: **p=0.007, *p=0.01.

KCNQ2/3	τ deact, -120 mV (ms)	τ deact, -110 mV (ms)	τ deact, -100 mV (ms)	τ deact, -90 mV (ms)	τ deact, -80 mV (ms)
**Ctrl**	13 ± 1.0 (n =5)	18 ± 1.0 (n =5)	25 ± 1.0 (n =5)	36 ± 1.6 (n =5)	52 ± 3.4 (n =5)
**Cocktail**	27 ± 2.3 (n =5) **	38 ± 3.4 (n =5) **	54 ± 5.0 (n =5) **	77 ± 7.1 (n =5) **	92 ± 4.7 (n =5) ***

Supplementary Table 33. Summary of effects of cocktail on KCNQ2/3 channel deactivation kinetics.

Statistics versus same channel in absence of cocktail: ***p=0.0003, **p=0.002.

KCNQ2/3	Normalized tail current $V_{0.5}$ (mV)	Non-normalized tail current $V_{0.5}$ (mV)	Slope (mV)
**Ctrl**	-48.0 ± 0.7 (n =31)	-47.6 ± 2.2 (n =31)	7.5 ± 0.8 (n =31)
**10 µM Retigabine**	-61.4 ± 1.1 (n =9) ****	-61.8 ± 4.8 (n =9) *	6.7 ± 1.1 (n =9)
**10 µM Retigabine 500 µM IVA**	-79.8 ± 0.9 (n =6) ****	-79.8 ± 0.9 (n =6) ****	7.5 ± 1.2 (n =6)
**10 µM Retigabine 30 µM MTX**	-104.6 ± 1.5 (n =6) ****	-100.9 ± 5.9 (n =6) ***	7.3 ± 1.2 (n =6)
**10 µM Retigabine 30 µM MTX 500 µM IVA**	n.d. (n =8)	n.d. (n =8)	n.d. (n =8)

Supplementary Table 34. Summary of Effects of MTX, IVA and retigabine combinations on KCNQ2/3 channels.

Statistics versus same channel in absence of MTX and IVA: ****p<0.0001, ***p=0.0003, *p=0.02. Values indicate mean ± SEM. n.d., not determined.

KCNQ2/3	Normalized tail current $V_{0.5}$ (mV)	Non-normalized tail current $V_{0.5}$ (mV)
**Ctrl**	-38.7 ± 0.7 (n =13)	-37.9 ± 2.2 (n =13)
**1 µM Retigabine**	-41.9 ± 0.7 (n =8) *	-41.7 ± 2.3 (n =8)
**1 µM Retigabine 1 µM MTX 1 µM IVA**	-54.3 ± 1.6 (n =8) ****	-49.8 ± 4.7 (n =8) *
**1 µM Retigabine 10 µM MTX 10 µM IVA**	-57.8 ± 0.9 (n =8) ****	-55.9 ± 3.5 (n =8) ***

Supplementary Table 35. Summary of Effects of low-dose retigabine, MTX and IVA on KCNQ2/3 channels.

Statistics versus same channel in absence of retigabine, MTX and IVA: ****p<0.0001, ***p=0.0009, **p=0.004, *p=0.05. Values indicate mean ± SEM.

KCNQ2	Normalized tail current $V_{0.5}$ (mV)	Non-normalized tail current $V_{0.5}$ (mV)
**Ctrl**	-46.5 ± 0.7 (n =7)	-46.5 ± 5.9 (n =7)
**1 µM Retigabine**	-51.7 ± 0.7 (n =7)	-51.6 ± 6.6 (n =7) ***
**1 µM Retigabine 1 µM MTX 1 µM IVA**	-56.1 ± 0.6 (n =7)	-56.4 ± 5.4 (n =7) ****
**1 µM Retigabine 10 µM MTX 10 µM IVA**	-63.7 ± 0.7 (n =7)	-64.1 ± 6.0 (n =7) ****
**10 µM Retigabine 30 µM MTX 500 µM IVA**	n.d.	n.d.

Supplementary Table 36. Summary of Effects of retigabine, MTX and IVA on KCNQ2 channels.

Statistics versus same channel in absence of retigabine, MTX and IVA: ****p<0.0001, ***p=0.0002.

KCNQ3*	Normalized tail current $V_{0.5}$ (mV)	Non-normalized tail current $V_{0.5}$ (mV)
**Ctrl**	-50.8 ± 2.1 (n =11)	-53.5 ± 8.6 (n =11)
**1 µM Retigabine**	-61.9 ± 2.2 (n =11) **	-63.9 ± 7.7 (n =11)
**1 µM Retigabine 1 µM MTX 1 µM IVA**	-74.4 ± 2.4 (n =11) ****	-76.5 ± 8.0 (n =11)
**1 µM Retigabine 10 µM MTX 10 µM IVA**	-91.4 ± 2.6 (n =11) ****	-91.6 ± 8.7 (n =11) **
**10 µM Retigabine 30 µM MTX 500 µM IVA**	n.d.	n.d.

Supplementary Table 37. Summary of Effects of retigabine, MTX and IVA on KCNQ3* channels.

Statistics versus same channel in absence of retigabine, MTX and IVA: ****p<0.0001, **p=0.005.

KCNQ4	Normalized tail current $V_{0.5}$ (mV)	Non-normalized tail current $V_{0.5}$ (mV)
**Ctrl**	-7.1 ± 1.5 (n =6)	-7.2 ± 10.5 (n =6)
**1 µM Retigabine**	-9.7 ± 1.1 (n =6)	-10.2 ± 7.5 (n =6)
**1 µM Retigabine<br>1 µM MTX<br>1 µM IVA**	-12.8 ± 1.1 (n =6) *	-13.2 ± 6.4 (n =6)
**1 µM Retigabine<br>10 µM MTX<br>10 µM IVA**	-17.4 ± 1.7 (n =6) **	-18.6 ± 5.6 (n =6)
**10 µM Retigabine<br>30 µM MTX<br>500 µM IVA**	-39.8 ± 3.0 (n =6) ****	-37.3 ± 3.2 (n =6) *

Supplementary Table 38. Summary of Effects of retigabine, MTX and IVA on KCNQ4 channels.

Statistics versus same channel in absence of retigabine, MTX and IVA: ****p<0.0001, **p=0.004, *p=0.05.

KCNQ5	Normalized tail current $V_{0.5}$ (mV)	Non-normalized tail current $V_{0.5}$ (mV)
**Ctrl**	-41.7 ± 0.9 (n =10)	-40.3 ± 2.4 (n =10)
**1 µM Retigabine**	-46.0 ± 0.9 (n =6) **	-45.8 ± 2.8 (n =6)
**1 µM Retigabine<br>1 µM MTX<br>1 µM IVA**	-49.5 ± 0.5 (n =6) ****	-49.1 ± 2.4 (n =6) *
**1 µM Retigabine<br>10 µM MTX<br>10 µM IVA**	-54.6 ± 0.6 (n =6) ****	-53.2 ± 2.8 (n =6) **
**10 µM Retigabine<br>30 µM MTX<br>500 µM IVA**	-61.4 ± 0.9 (n =4) ****	-60.6 ± 2.8 (n =4) ***

Supplementary Table 39. Summary of Effects of retigabine, MTX and IVA on KCNQ5 channels.

Statistics versus same channel in absence of retigabine, MTX and IVA: ****p<0.0001, ***p=0.0009, **p=0.004, *p=0.05.
